# Effects of formaldehyde exposure on the development of pulmonary fibrosis induced by bleomycin in mice

**DOI:** 10.1016/j.toxrep.2018.03.016

**Published:** 2018-04-05

**Authors:** Mayara Peres Leal, Robson Alexandre Brochetti, Aline Ignácio, Niels Olsen Saraiva Câmara, Renata Kelly da Palma, Luis Vicente Franco de Oliveira, Daniela de Fátima Teixeira da Silva, Adriana Lino-dos-Santos-Franco

**Affiliations:** a Post Graduate Program in Biophotonics Applied to Health Sciences, University Nove de Julho (UNINOVE), São Paulo, Brazil; b Department of Immunology, University of São Paulo, São Paulo, Brazil; c Post Graduate Program in Science of rehabilitation, University Nove de Julho (UNINOVE), São Paulo, Brazil

**Keywords:** Formaldehyde exposure, Pulmonary fibrosis, Lung inflammation, Cytokines, Lung elastance, Collagen production

## Abstract

Environmental and Occupational pollution has been extensively studied because of its serious implications on the human health. Formaldehyde (FA) is a pollutant widely employed in several industries and also in anatomy, pathology and histology laboratories. Studies have shown the correlation between FA exposure and development or worsening of asthma. However, the effect of FA exposure on the pulmonary fibrosis (PF) is unknown. PF is a progressive and chronic lung disease with high incidence and considerable morbidity and mortality. Few studies have shown a worsening of PF after pollutants exposure such as ozone and nitrogen dioxide. Therefore, our objective was to assess the effects of FA on the PF. Male mice C57BL6 were treated or not with bleomycin (1,5 U/kg) and exposed or not to FA inhalation (0.92 mg/m^3^, 1 h/day, 5 days/week during 2 weeks). Non-manipulated mice were used as control. Our data showed that FA exposure in fibrotic mice increased the number of granulocytes in the bronchoalveolar lavage followed by elevated levels of interleukin 1 beta and interleukin 17. In addition, FA exposure in fibrotic mice enhanced the gene expression of C-X-C motif chemokine ligand 1 (CXCL1) and tumor necrosis factor alpha (TNF-α) in the lung. We also showed an increase in the collagen production, while lung elastance was reduced. **No** differences were found in the mucus production, oedema and interstitial thickening in the lung tissue of fibrotic mice after FA exposure. In conclusion our study showed that FA exposure aggravates the lung neutrophils influx and collagen production, but did not alter the lung elastance, mucus production, oedema and interstitial tickening. This work contributes to understand the effects of pollution in the development of PF.

## Highlights

• FA exposure aggravates the development of PF.
• FA exposure intensifies the collagen production into the lung worsening the PF.
• FA exposure exacerbates the lung cellular recruitment by CXCL1 and IL-17 mechanisms-involved.

## Graphical abstract

**Figure:**
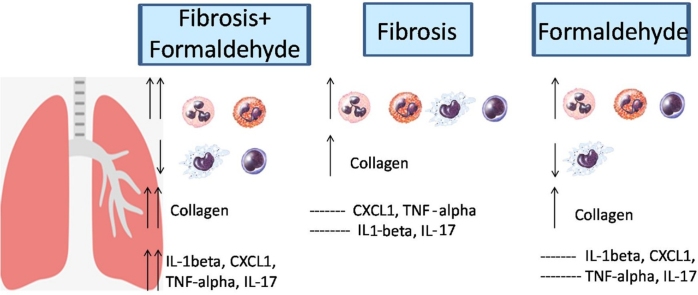

## 1 Introduction

Formaldehyde (FA) is a pollutant widely employed in several industries and also in anatomy, pathology and histology laboratories. Studies have shown the correlation between exposure to FA and development or worsening of asthma [1], [2], [3], [4], [5], [6]. In addition, FA exposure during the pregnancy causes changes in the immune system of offspring, culminating to impaired defenses against infectious or allergic stimulus by fetal programming mechanisms [7], [8]. Thus, the effects of FA have been studied extensively; however, the link between FA and pulmonary fibrosis (PF) is not still established.

PF is a progressive and chronic condition, whose etiology is unknown and the incidence is high [9]. It is characterized by lung inflammation and loss of the original architecture of the lung due to excessive and disorganized expression and deposition of collagen and extracellular matrix leading to lung remodeling and respiratory failure [10], [11]. The Pulmonary fibrosis can be developed experimentally by bleomycin administration. Bleomycin is an antineoplastic agent that can cause lung toxicity. This toxicity is related to several inflammatory mediators including transforming growth factor-beta (TGF-beta) and tumor necrosis factor-alpha (TNF-alpha), chemokines among others, which cause severe lung fibrosis as side effect.

The accumulation of vascular exudate and inflammatory cells within the alveolar space causes epithelial injury. These exudates increase the proliferation of resident fibroblasts and their differentiation into myofibroblasts [12]. The balance between inflammatory and anti-inflammatory cytokines is very important for the control of the disease. In this context, transforming growth factor- beta (TGF-beta) is produced by inflammatory cells inducing the differentiation of fibroblasts into myofibroblasts. After that, the synthesis and secretion of collagen is substantially increased causing lung remodeling [13]. In addition, it is an important sign for induction of interleukin 17 (IL-17), which amplifies the development of lung fibrosis [14]. It is well established that interleukin 17 (IL-17) promotes the recruitment of neutrophils and might be responsible by the mortality in patients with PF [15]. Similarly, CXC chemokine superfamily, such as CXCL1 is also produced by alveolar macrophages and it plays a central role in the PF regulating the neutrophil influx [16]. It has been shown that the level of CXCL1 into the lung correlates with neutrophil inﬂux [17]. Interleukin 6 (IL-6) is an inflammatory cytokine produced by many cells and its inhibition reduces the lung fibrosis in murine model [18]. Anti-inflammatory cytokines including interleukin 10 (IL-10) and interferon gamma (IFN-γ) are increased in patients with PF and may be used as a biomarker or a therapeutic target because its anti-fibrotic effects [19].

There are few studies in the literature evaluating the effects of pollutants on the worsening of PF. Epidemiological study correlated interstitial lung diseases with inhaled agents including cigarette smoke, organic antigens and wood dusts [20]. Moreover, Johannson et al. [21] showed that exposure to ozone and nitrogen dioxide induced deleterious effects on the PF. Such studies suggest that the pollution may contribute to the worsening of PF. Thus, additional studies are needed. In this context, we evaluated the effects of FA exposure, since it is an important occupational and environmental pollutant. Our study prioritized the effects of FA on the cell recruitment, release and gene expression of cytokines, collagen and mucus production, oedema, interstitial thickening and lung elastance.

## 2 Material and methods

### 2.1 Animals

The animals were obtained from the University Nove de Julho and the experiments were approved by the Animal Care Committee University Nove de Julho (CoEP-UNINOVE, AN0038/2014). Male C57BL/6 mice were maintained in light and temperature-controlled room (12/12-hour light-dark cycle, 21 ± 2 °C), with free access to food and water.

### 2.2 Experimental model of pulmonary fibrosis (PF)

The animals were subjected to a single bleomycin injection by orotracheal route (1.5 U/kg) under soft anesthesia (ketamine-xylazine) [22], [23].

### 2.3 Exposure to formaldehyde inhalatio

The animals were exposed or not to FA inhalation (0.92 mg/m^3^, 1 h/day, 5 days/week during 2 weeks) seven days after the PF induction. The dose as well as the time of FA exposition was based on previous studies [7], [8].

### 2.4 Groups of study and experimental design

The animals were divided into 4 groups (n = 6 animals) and they were killed by sectioning the abdominal aorta under deep anesthesia with ketamine-xylazine by intraperitoneal route (100 mg/kg and 20 mg/kg, respectively) 24 h after the last FA inhalation. Below are listed the experimental groups:

Fibrosis + FA vehicle inhalation (F group): Animals submitted to bleomycin injection and exposed to FA vehicle.

Formaldehyde inhalation (FA group): Animals submitted to bleomycin vehicle injection and exposed to FA inhalation.

Fibrosis + Formaldehyde inhalation (F + FA group): Animals submitted to bleomycin injection and exposed to FA inhalation.

Basal (B group): Non-manipulated animals.

### 2.5 Evaluation of lung cell influx

The number of cells migrated to the bronchoalveolar lavage (BAL) was determined according to Maiellaro et al. [7]. The total cells as well as the differential cells were determined by microscopy.

### 2.6 Quantification of cytokines generated by BAL cells

Cytokines levels were determined in the BAL supernatant samples according to the manufacturer's specifications using ELISA kits purchased from Biolegend (San Diego, USA). Results were expressed as pg of cytokine produced per ml.

### 2.7 Quantification of gene expression of cytokines

Gene expression of cytokines was determined according to Silva Macedo et al. [24]. RT-PCR was performed using Taqman real-time PCR assay (Applied Biosystem, USA) for the following molecules: TNF-α: (Mm00443258_m1), IL-1β (Mm00434228_m1), IL-17(Mm01189488_m1), CXCL1 (Mm04207460_m1) and, TGF-β (Mm01178820_m1). Sequence Detection Software 1.9 (SDS) was used for the analysis and mRNA expression was normalized to HPRT expression.

### 2.8 Evaluation of collagen production in the lung tissue

Histological analyses were performed according to Miranda da Silva et al. [1] and it was evaluated by 1 blinded observer. Lung tissues were stained with Picrossirius and the analyses were performed by image using the Image Pro plus program.

### 2.9 Determination of lung elastance

Lung elastance is a measure of the tendency of a hollow organ to recoil toward its original dimensions upon removal of a distending or compressing force and it was determined on anesthetized mice using a volumetric ventilator (MV215, Montevideo, UY) in open as well as closed chest [22]. Briefly, mice were anesthetized with a ketamine and xylazine, tracheostomized, and subjected to conventional ventilation with a quasi-sinusoidal flow pattern with a tidal volume of 10 ml/kg of mouse body weight, a frequency of 100 breaths/min, and a positive end expiratory pressure of 2 cmH_2_O. Flow and pressure signals from the transducers were analogically lowpass filtered, sampled and stored for subsequent analysis.

### 2.10 Evaluation of mucus production, oedema and interstitial tissue thickening

Histological analyses were determined by histomorphometric technique by 1 blinded observer. Below are described the scores used for the evaluations.

#### 2.10.1 Mucus accumulation

0, absent; 1, discontinuous presence on the epithelial surface; 2, presence of goblet cell metaplasia in the bronchial epithelium and continuous epithelial surface; 3 presence of goblet cell metaplasia in the bronchial epithelium and a layer of thick mucus on the epithelial surface; 4, a partial obstruction of the bronchiolar lumen by a layer of mucus.

#### 2.10.2 Oedema

0, absent; 1, minimal presence of plasma in the interstitial tissue; 2, presence of large amounts of plasma in the interstitial tissue; 3, presence of plasma and red blood cells in the interstitial space; 4, abundant presence of cells, plasma and red blood cells in the interstitial space.

#### 2.10.3 Interstitial tissue thickening

0, absence; 1, increased cellularity in interstitial tissue; 2, increased cellularity in interstitial tissue and tissue thickness in up to one cell layer; 3, increased cellularity in interstitial tissue and tissue thickness in up to two cell layers; 4 increased cellularity in the interstitial tissue and tissue thickness with visible presence of collagen fibers.

### 2.11 Statistical analyses

Data were expressed as the means ± SEM, and comparisons among the experimental groups were analyzed by one-way ANOVA followed by the Student´s Newman-Keuls test for multiple comparisons using the GraphPad software V.5. P-values less than 0.05 were considered statistically significant. These analyses were performed considering the differences among FA, Fibrosis and Basal group as well as if FA exposure could aggravate the fibrosis parameters.

## 3 Results

### 3.1 FA exposure increased the number of granulocytes recruited in the BAL of fibrotic mice

**Fig. 1:**
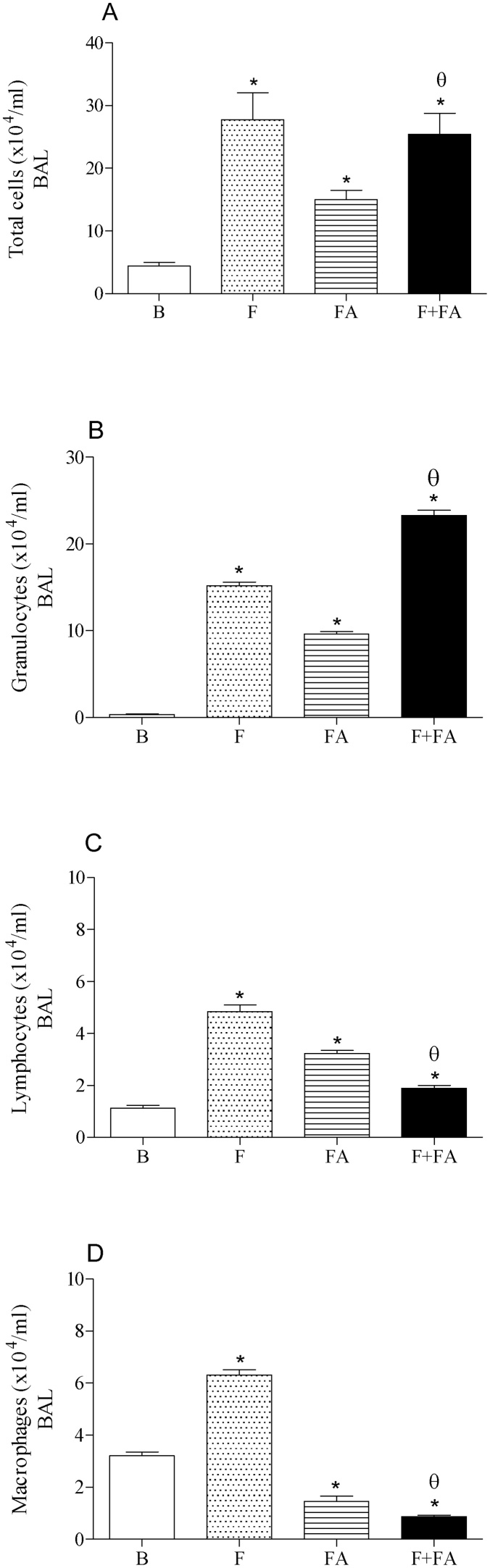
**FA Exposure increases the number of granulocytes in the BAL of fibrotic mice.** Groups of mice were submitted to bleomycin injection (1.5 U/kg, orotracheal route) and after 7 days were exposed to FA inhalation (0.75 ppm, 1 h/day, 15 days). Non-manipulated animals were used to basal parameters. After 24 h of last exposure to FA or 14 days after fibrosis development, the BAL was performed. Data represent the mean ± SEM of 6 animals. *P < 0.05 compared to the B group; ^θ^*P* < 0.05 compared to the F group; ^δ^*P* < 0.05 compared to the FA group.

Fig. 1 (panel A) showed elevated number of cells into the BAL after bleomycin injection (F group; 27.75 ± 2.8 × 10^4^ cells/ml, P < 0.0001) as well as after FA exposure (FA group; 15.0 ± 0.11 × 10^4^ cells/ml, P < 0.05) when compared to the Basal group (B group; 4.42 ± 0.3 × 10^4^ cells/ml). On the other hand, FA exposure in fibrotic mice did not alter the number of cells recruited into the BAL in relation to F group. In addition, we observed elevated number of cells in F + FA group (25.40 ± 2.45 × 10^4^ cells/ml) when compared to the B (P < 0.0001) and FA (P < 0.05) groups.

In panel B we can observe elevated number of granulocytes migrated into the BAL of F (15.18 ± 1.48 × 10^4^ cells/ml), FA (9.60 ± 0.95 × 10^4^ cells/ml) and F + FA (23.30 ± 2.34 × 10^4^ cells/ml) groups in relation to B group (0.34 ± 0.030 × 10^4^ cells/ml; P < 0.0001). In addition, FA exposure in fibrotic mice increased significantly the number of granulocytes in the BAL in relation to FA and F groups (P < 0.0001).

In panel C we also observed elevated number of lymphocytes in the BAL of F (4.85 ± 0.51 × 10^4^ cells/ml), FA (3.22 ± 0.33 × 10^4^ cells/ml) and F + FA (1.90 ± 0.20 × 10^4^ cells/ml) groups when compared to the B group (1.12 ± 0.10 × 10^4^ cells/ml, P < 0.0001). Moreover, a reduction in the number of lymphocytes was observed in the group F + FA in relation to F and FA groups (P < 0.0001).

As can be observed in panel D, the injection of bleomycin elicited an increase in the macrophages recruited in the BAL (F group, 6.3 ± 0.65 × 10^4^ cells/ml) when compared to non-treated mice (B group 3.2 ± 0.28 × 10^4^ cells/ml, P < 0.0001). On the other hand, we observed reduced number of macrophages in FA group (1.45 ± 0.15 × 10^4^ cells/ml) in relation to B group (P < 0.0001). Moreover, an additional reduction was observed in F + FA group (0.86 ± 0.077 × 10^4^ cells/ml) when compared to the F (P < 0.0001) and FA (P < 0.05) groups

### 3.2 FA exposure elevated the levels of IL-1beta and IL-17 in the supernatant of BAL of fibrotic mice

**Fig. 2:**
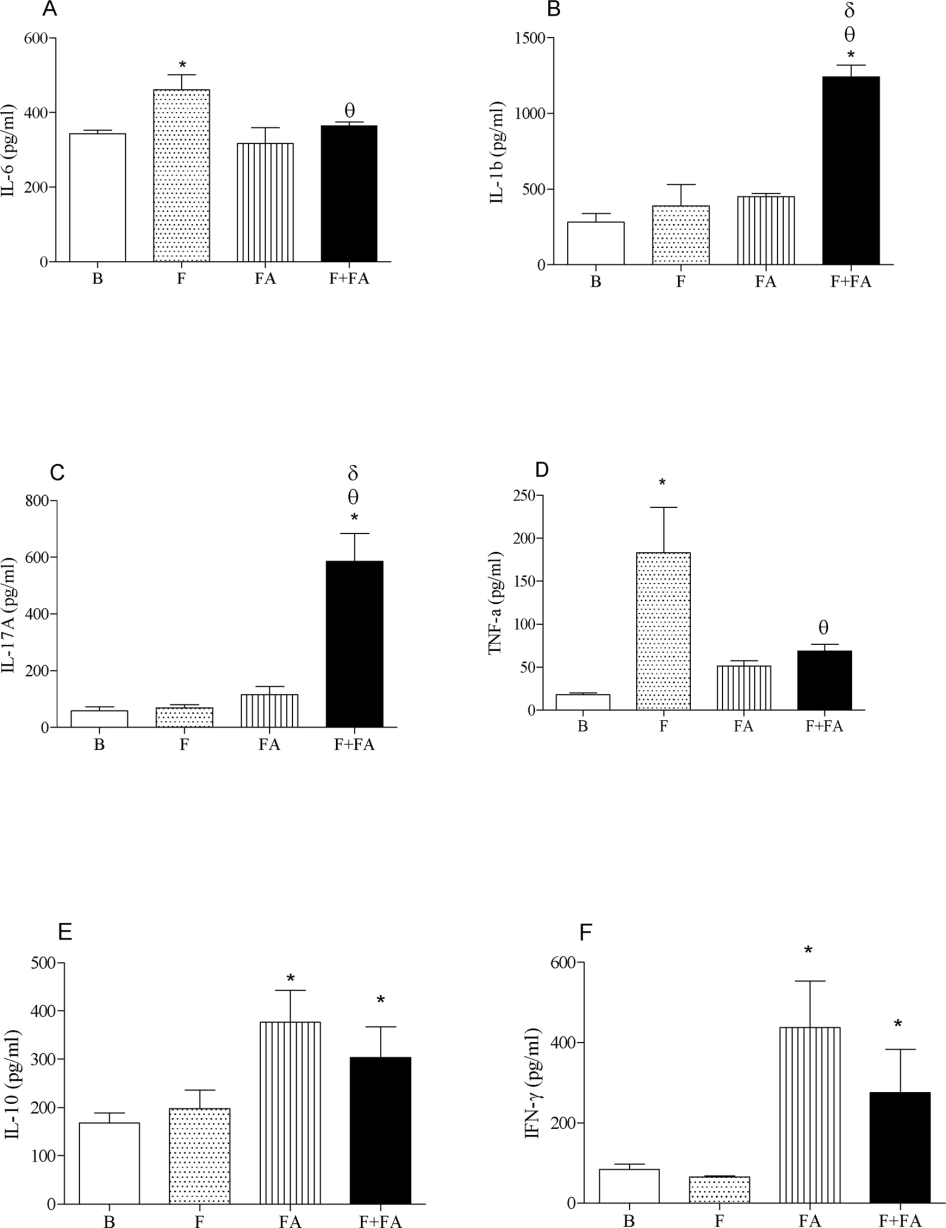
**FA exposure elevates the levels of IL-1beta and IL-17 in the supernatant of BAL of fibrotic mice.** Groups of mice were submitted to bleomycin injection (1.5 U/kg, orotracheal route) and after 7 days were exposed to FA inhalation (0.75 ppm, 1 h/day, 15 days). Non-manipulated animals were used to basal parameters. After 24 h of last exposure to FA or 14 days after fibrosis development, the cytokines were determined. Data represent the mean ± SEM of 6 animals. *P < 0.05 compared to the B group; ^θ^*P* < 0.05 compared to the F group; ^δ^*P* < 0.05 compared to the FA group.

Fig. 2 (Panel A) shows reduced level of IL-6 in F + FA group (364.4 ± 36.8 pg/ml) when compared to the F group (460.6 ± 45 pg/ml, P < 0.05). We also observed increased level of IL-6 in F group in relation to B group (343.3 ± 33.5 pg/ml; P < 0.05).

In panels B and C we showed elevated levels of IL-1beta and IL-17 respectively in F + FA group (1241.2 ± 114 and 585.8 ± 58 pg/ml) in relation to B (282.3 ± 27 pg/ml and 58.5 ± 6 pg/ml; P < 0.0001), F (388.0 ± 37.7 and 69.0 ± 7.11 pg/ml; P < 0.0001) and FA groups (450.3 ± 42.1 and 115.6 ± 12.2 pg/ml; P < 0.0001). Moreover, no differences were observed among B, F and FA groups.

We also observed in panel D reduced levels of TNF-alpha in F + FA group (68.75 ± 7 pg/ml) in relation to F group (183.0 ± 25.4 pg/ml, P < 0.0001) and did not differ from FA group (51.2 ± 50 pg/ml). In addition, elevated levels of TNF-alpha were observed in F group when compared to the B group (18.0 ± 1.69 pg/ml, P < 0.0001)

In panels E and F we can observe elevated level of IL-10 and IFN-gamma respectively in FA (376.8 ± 80.3; 437.2 ± 120 pg/ml) and F + FA groups (303.5 ± 82; 275.5 ± 145 pg/ml) in relation to B group (167.6 ± 25; 84 ± 7.6 pg/ml; P < 0.05). No differences were observed between B and F groups.

### 3.3 FA exposure elevated the gene expression of CXCL1 and TNF-alpha in the lung tissue of fibrotic mice

**Fig. 3:**
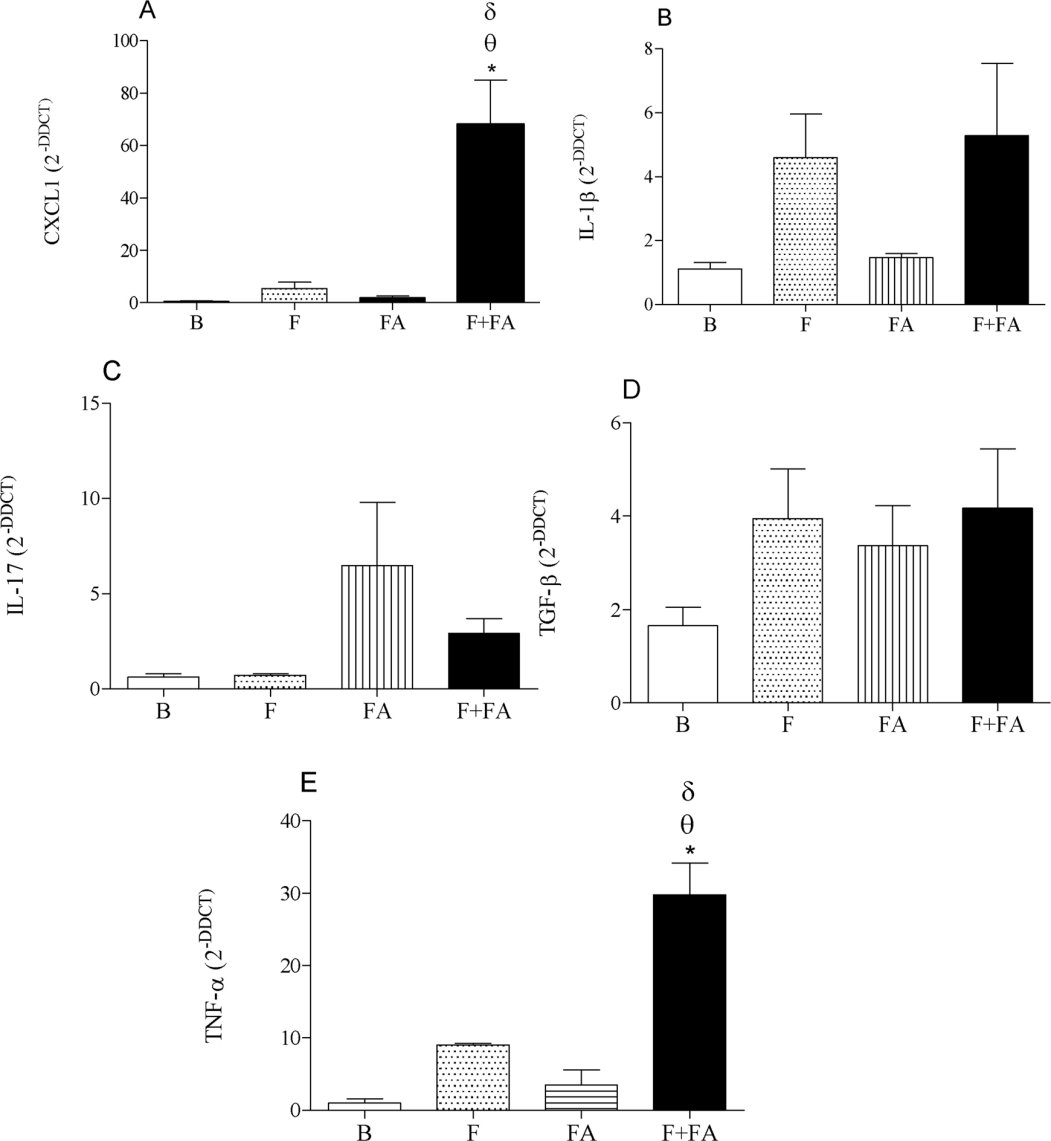
**FA exposure elevates the gene expression of CXCL1 and TNF-alpha in the lung tissue of fibrotic mice.** Groups of mice were submitted to bleomycin injection (1.5 U/kg, orotracheal route) and after 7 days were exposed to FA inhalation (0.75 ppm, 1 h/day, 15 days). Non-manipulated animals were used to basal parameters. After 24 h of last exposure to FA or 14 days after fibrosis development, the gene expression of cytokines was determined. Data represent the mean ± SEM of 6 animals. *P < 0.05 compared to the B group; ^θ^*P* < 0.05 compared to the F group; ^δ^*P* < 0.05 compared to the FA group.

Fig. 3 (Panels A and E) shows that exposure to FA in fibrotic mice increased the gene expression of CXCL1 (F + FA group 67.9 ± 14) and TNF-alpha (29.73 ± 5) when compared to the F (5.50 ± 0.56 and 9.06 ± 0.77; P < 0.05), FA (1.92 ± 0.19 and 3.52 ± 0.44; P < 0.05) and B groups (0.46 ± 0.0038 and 1.049 ± 0.13; P < 0.05).

In panels B, C and D no differences were observed among the groups of study.

### 3.4 FA exposure increased the collagen production in the lung tissue of fibrotic mice

**Fig. 4:**
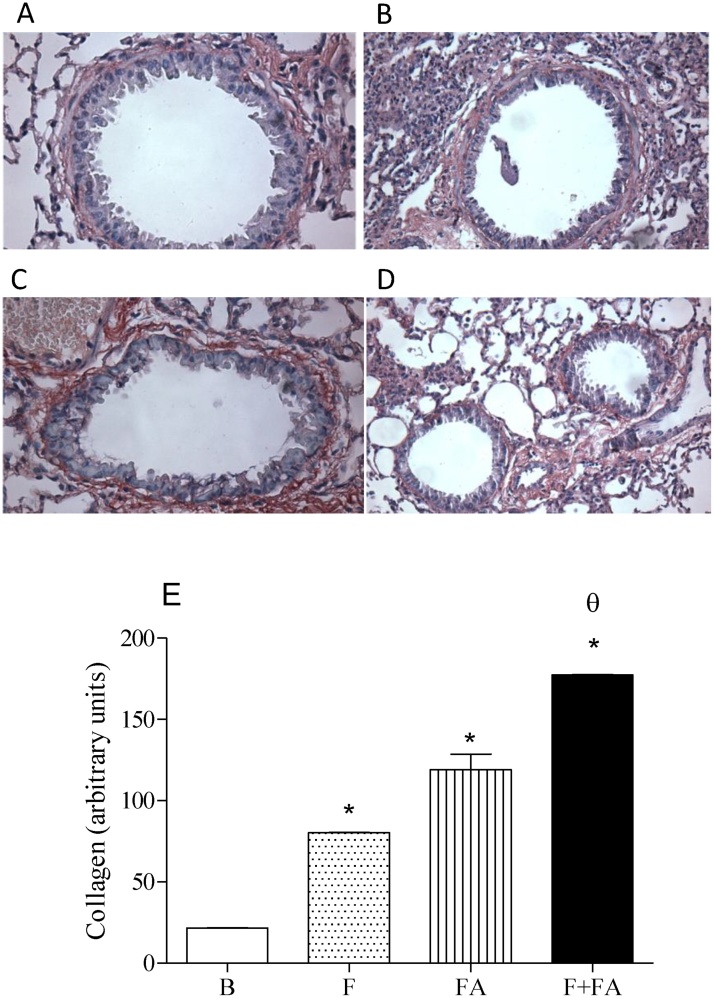
**FA exposure increases the collagen production of fibrotic mice**. Groups of mice were submitted to bleomycin injection (1.5 U/kg, orotracheal route) and after 7 days were exposed to FA inhalation (0.75 ppm, 1 h/day, 15 days). Non-manipulated animals were used to basal parameters. After 24 h of last exposure to FA or 14 days after fibrosis development, the collagen production was evaluated. Data represent the mean ± SEM of 6 animals. *P < 0.05 compared to the B group; ^θ^*P* < 0.05 compared to the F group; ^δ^*P* < 0.05 compared to the FA group. Panels A, B, C and D represent the basal, fibrosis, FA and FA + fibrosis groups respectively.

Fig. 4 (Panels A–D) shows the collagen production of B, F, FA and F + FA groups respectively. The representative graph of these figures can be observed in panel E. It shows that FA exposure in fibrotic mice (F + FA group 177.3 ± 16.60) increased the collagen production in relation to F (80.2 ± 7.23; P < 0.0001), FA (119.0 ± 12.8; P < 0.0001) and B groups (21.5 ± 1.88; P < 0.0001). We can also observe that both F and FA groups increased the collagen production when compared to the B group (P < 0.0001).

### 3.5 FA exposure reduced static elastance (Est) without alter dynamic elastance (Edyn) in fibrotic mice

**Fig. 5:**
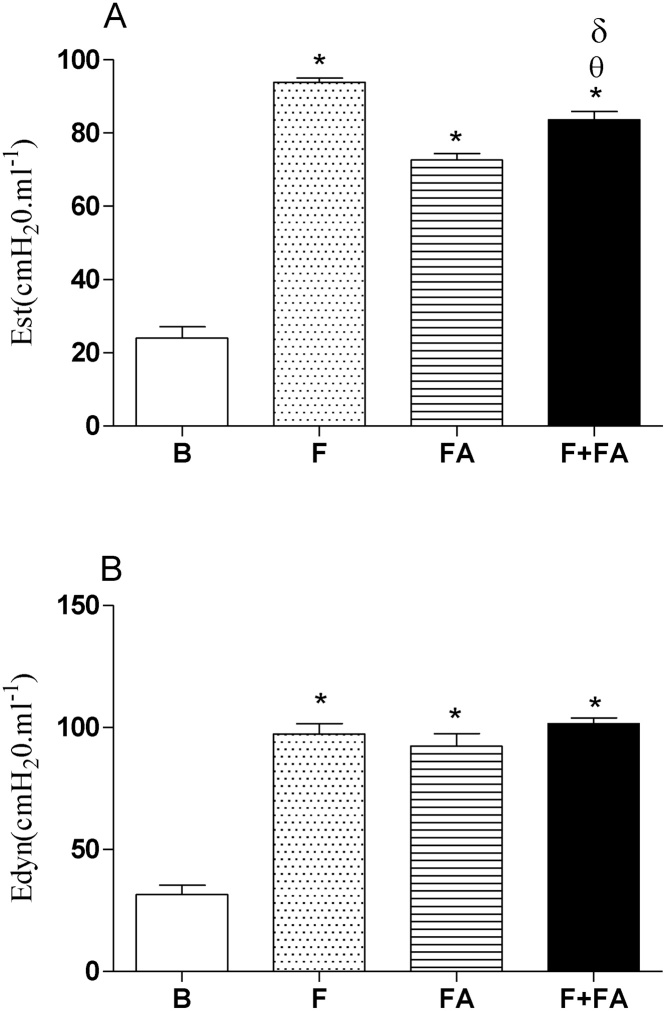
**FA exposure reduced the static lung elastance while did not alter the dynamic lung elastance of fibrotic mice.** Groups of mice were submitted to bleomycin injection (1.5 U/kg, orotracheal route) and after 7 days were exposed to FA inhalation (0.75 ppm, 1 h/day, 15 days). Non-manipulated animals were used to basal parameters. After 24 h of last exposure to FA or 14 days after fibrosis development, the lung elastance was evaluated. Data represent the mean ± SEM of 6 animals. *P < 0.05 compared to the B group; ^θ^*P* < 0.05 compared to the F; ^δ^*P* < 0.05 compared to the FA group.

Fig. 5 (Panel A) shows elevated Est in F (98.8 ± 9), FA (72.6 ± 6.75) and F + FA (83.6 ± 8.7) groups in relation to B group (24.02 ± 2.22; P < 0.0001). In addition, we can note decreased Est in F + FA group when compared to the F group (P < 0.0001). On the other hand, increased Est was observed in F + FA group in relation to FA group (P < 0.0001).

In panel B we can observe that no differences were observed among F, FA and F + FA groups. On the other hand, we had an elevated Edyn in F (97.3 ± 9.32), FA (92.26 ± 9.2) and F + FA (101.5 ± 9.9) groups when compared to the B group (31.4 ± 3.19; P < 0.0001).

### 3.6 FA exposure in fibrotic mice did not alter the mucus production, oedema and interstitial thickening

In Table 1 we can observe that both groups, F and F + FA, showed an elevated mucus production, oedema, and interstitial tickening in relation to B group (P < 0.0001). Moreover, we noted that mucus production, oedema and interstitial tickening of F + FA group did not differ from F and FA groups.

**Table 1:** The effects of formaldehyde on the histopathological characteristics during lung fibrosis. Groups of mice were submitted to bleomycin injection (1.5 U/kg, orotracheal route) and after 7 days were exposed to FA inhalation (0.75 ppm, 1 h/day, 15 days). Non-manipulated animals were used to basal parameters. After 24 h of last exposure to FA or 14 days after fibrosis development, the lung mucus, oedema and interstitial tickening were evaluated. Data represent the mean ± SEM of 6 animals. *P < 0.05 compared to the B group.

**Groups**	**Mucus**	**Oedema**	**Interstitial tickening**
B	0,33	0,16	0,16
FA	1,33*	1,33*	1,50*
F	2,50*	2,25*	2,75*
F + FA	2,20*	2,60*	2,00*

## 4 Discussion

Using an experimental model of PF induced by bleomycin, we showed the effects of FA exposure in the inflammatory and fibrotic parameters. This experimental model is well established and it reproduces the physiopathology of human disease, which is characterized by lung inflammation and loss of the original architecture of the lung due to excessive and disorganized expression and deposition of collagen and extracellular matrix [10], [11].

The effects of pollution in the PF are still unknown. Few studies evaluated the impact of pollutants in the course of PF. In this context, epidemiological study correlated interstitial lung diseases with inhaled agents including cigarette smoke, organic antigens and wood dusts [20]. Moreover, Johannson et al. [21] showed that exposure to ozone and nitrogen dioxide caused deleterious effects on the PF. Thus, these studies suggest that pollution may contribute to the worsening of this disease.

Considering that in earlier studies we showed that FA exposure induces lung inflammation [4], interferes in the development of asthma [3], [25], and alters the immune system of offspring [7], [8], we investigated its impact in the course of PF, which is not established. The protocol of FA inhalation used here was based in the previous studies [8], [7]. It is important to mention that the dose of FA used in the present study do not cause effects in the human health (OSHA).

PF is characterized by progressive lesion of the pulmonary parenchyma, inflammatory infiltrate and fibrosis in the interstitial space. It is known that granulocytes (neutrophils) play an important role in the epithelial injury [23]. In addition, mediators released by neutrophils contribute to the proliferation of resident fibroblasts as well as their differentiation into myofibroblasts (activated fibroblasts secreting large amounts of collagen) [12].

First we evaluated the lung inflammation by quantification of cells recruited in the BAL. Our results showed that FA exposure in fibrotic mice caused marked increase in the granulocytes recruited in the BAL followed by reduced number of macrophages and lymphocytes; despite of the number of total cells were not modified.

This result is important if we consider the role of neutrophils in the development and worsening of PF. Moore et al. [23] showed that patients with PF develop inflammatory episodes that were related to the progression of the disease. Moreover, apoptosis can regulate the magnitude of the inflammatory response as well as the resolution process. Inflammatory mediators such as interferon alpha, interleukins 2 and 6, leukotrienes among others may inhibit neutrophils apoptosis, extending their survival and amplifying the lung inflammation [26]. Thus, we might hypothesize that this elevated number of neutrophils, eventually, could be a reflex of a prolonged survival occasioned by FA exposure.

The balance between pro-inflammatory and anti-inflammatory cytokines is very important to lung homeostasis. In order to explain the elevated number of neutrophils, we investigated some important cytokines involved in the PF. It is know that IL-17 promotes the neutrophils influx into the airways and might be a responsible by the mortality of patients with PF [15]. Interleukin 6 (IL-6) is an inflammatory cytokine and its inhibition is linked with reduced lung fibrosis [18]. TNF-α, interleukins 6, 1 beta, 8 among others, are also critical in the development of PF and mainly generated by macrophages.

Anti-inflammatory cytokines including IL-10 and interferon gamma (IFN-γ) are increased in patients with PF and may be used as a biomarker or a therapeutic target due their anti-fibrotic effects [19].

Based in the role of the main cytokines released during PF, our results showed that FA exposure increased the levels of IL-1beta and IL-17, while did not alter IL-10 and IFN-gamma. Recognizing that both, IL-1beta and IL-17 are involved in the neutrophils recruitment, and these cells consequently contribute to a worse of PF, we assume that FA exposure had deleterious effects in the course of PF. In addition, neutrophils can generate IL-1beta and IL-17, amplifying the lung inflammation. On the other hand, FA exposure did not modify anti-fibrotic cytokines (IL-10 and IFN-gamma). Such cytokines have protective effects during PF. Moreover, FA exposure decreased the levels of TNF-alpha and this reduction might be a reflex of reduced number of macrophages.

Subsequently we investigated the role of FA exposure in the gene expression of cytokines. We showed that FA exposure increased the gene expression of CXCL1, an important chemokine that regulates the neutrophil influx into inflamed tissue. In addition, our results showed that FA exposure elevated the gene expression of TNF-α, although we did not find this cytokine elevated in the supernatant of BAL. Probably, this discrepancy can be explain by the time point of analyses. We must consider that all of analyses were performed after 24 h the last FA exposure. Eventually, some alterations can appear later.

We analysed other inflammatory parameters including mucus production, oedema and interstitial tickening and FA did not cause an additional increment. Probably, this result reflects the maximum response developed by bleomycin.

Despite of the effects of FA in the lung inflammation, it is important to consider that many studies show that the biggest problem of PF is the collagen production and not necessary the inflammatory process. Considering the important role of collagen in the course of PF, we investigated whether FA exposure could aggravate the collagen production in fibrotic mice, and our data showed that FA exposure, in fact, elevated the collagen production in the lung leading to a worst of pathology. Curiously, the exacerbated collagen production observed in F + FA group did not cause additional reduction in the lung function. Eventually, bleomycin induced important alterations in the lung elastance, in order to FA was not able to cause an increment on this parameter.

The study of lung function includes spontaneous respiratory physiology as well as ventilatory support. Mathematical models are employed for the understanding of respiratory mechanics through the use of mechanical ventilators, both in clinical practice, eg in hospital ICUs, and in scientific research.

It is known that PF is characterized by changes in the lung microstructure due the collagen deposition. These structural changes, in turn, modify the gas exchange. Thus, changes in the lung mechanics can be assessed by means of airflow and lung capacity measurements. In this sense, we evaluated the lung elastance, defined as the property of resistance to deformation. Regarding elastance, it is important to understand the differences between static elastance (Est) and dynamic elastance (Edyn). For static elastance the volume variation refers to the static plateau pressure, while the dynamics the volume variation refers to the inspiratory peak pressure.

Our data showed that both bleomycin injection and FA exposure increased the lung elastance. This result for the fibrosis group is expected since the reduction in the lung function is peculiar to pulmonary fibrosis. Similar data were obtained by Gendron et al. [27], which determined the kinetics of fibrosis. These studies compared the total lung capacity in mice exposed to saline and bleomycin and they showed decreased in the total lung capacity in bleomycin-treated mice when compared to saline mice.

The increased lung elastance found in the FA group was surprising as well as the increase in the lung inflammation, collagen production and alveolar enlargement because the dose used in this study was very low and allowed for human exposure. Moreover, in earlier studies this dose of FA did not induce direct effects in the lung of pregnant rats, but it altered the immune system of offspring [8], [7]. We attribute such differences to the animal species used in both studies (rats and mice). The differences between rat and mice body weight could justify the harmful effects in mice.

In conclusion our study showed that FA exposure aggravates the lung neutrophils influx and collagen production, but did not alter the lung elastance, mucus production, oedema and interstitial tickening. This work contributes to understand the effects of pollution in the development of PF.

## Transparency document

Transparency document
